# ﻿Molecular and morphological evidence revalidates *Acrobrycontarijae* (Characiformes, Characidae) and shows hidden diversity

**DOI:** 10.3897/zookeys.1091.73446

**Published:** 2022-03-31

**Authors:** Yanina F. Briñoccoli, Sergio Bogan, Dahiana Arcila, Juan J. Rosso, Ezequiel Mabragaña, Sergio M. Delpiani, Juan Martín Díaz de Astarloa, Yamila P. Cardoso

**Affiliations:** 1 Laboratorio de Ictiofisiología y Acuicultura, Instituto Tecnológico Chascomús, Universidad Nacional de San Martin, Av. Intendente Marino km 8200 CC 164 7130, Chascomús, Buenos Aires, Argentina; 2 Fundación de Historia Natural “Félix de Azara”, Centro de Ciencias Naturales, Ambientales y Antropológicas, Universidad Maimónides, Hidalgo 775 piso 7, C1405BDB, Buenos Aires, Argentina; 3 Sam Noble Oklahoma Museum of Natural History and Department of Biology, University of Oklahoma, Norman, Oklahoma, 73072, USA; 4 Grupo de Biotaxonomía Morfológica y Molecular de Peces, Instituto de Investigaciones Marinas y Costeras (IIMyC), Facultad de Ciencias Exactas y Naturales, Universidad Nacional de Mar del Plata, Funes 3350, 7600 Mar del Plata, Argentina; 5 Laboratorio de Sistemática y Biología Evolutiva, Facultad de Ciencias Naturales y Museo, Universidad Nacional de La Plata. Paseo del Bosque S/N, B1900FWA, La Plata, Buenos Aires, Argentina; 6 Consejo Nacional de Investigaciones Científicas y Técnicas (CONICET), Buenos Aires, Godoy Cruz 2290, CABA, Argentina; 7 Departamento de Biología, Facultad de Ciencias Exactas y Naturales, Universidad Nacional de Mar del Plata, Funes 3250, 7600 Mar del Plata, Argentina

**Keywords:** Endorheic, freshwater fishes, La Plata River Basin, mitochondrial DNA, Stevardiinae

## Abstract

We conducted a revision of the Neotropical genus *Acrobrycon*. A previous study synonymized the species, *A.ipanquianus*, distributed from the western portion of the Amazon River to the north-western region of the La Plata River Basin, and *A.tarijae*, with type locality in the Lipeo River in Bolivia. We revisited this result by collecting new morphometric, meristic, and genetic data (*COI* mitochondrial gene) for 24 individuals distributed along La Plata River Basin in Argentina, and discussed our results in the context of multiple biogeographic processes of isolation in that basin. Our results revealed a more complex history of diversification and geographic distribution across *Acrobrycon* species than previously suspected, probably associated with multiple biogeographic processes of isolation in La Plata River Basin. We present new evidence that led us to reconsider the validity of *A.tarijae*, which is distinguishable from *A.ipanquianus* by the number of vertebrae (37–39 vs. 41–42) and pleural ribs (12–13 vs. 14). These results were also supported by our molecular analyses that revealed a genetic divergence >4% between *A.ipanquianus* and *A.tarijae*. We also identified two main genetic clusters within *A.tarijae*: the first cluster consisted of specimens from the Bermejo, Pilcomayo, Itiyuro and Juramento river basins (northern Argentina); and the second cluster included specimens from the southernmost basins, such as the Salí River in Tucumán, Cuarto River in the province of Cordoba and the Quinto River in the province of San Luis. Our results suggest that the genetic structure observed in *A.tarijae* is the result of the type of drainage (endorheic vs. exorheic) and geographical distance.

## ﻿Introduction

Among freshwater fishes, Characidae is the most diverse family of the order Characiformes with over 1180 valid species (Fricke et al. 2021). Phylogenetic relationships among characids based on reproductive, morphological and molecular characters have been largely controversial due to the taxonomic complexity of this family (Javonillo et al. 2010; Mirande 2010, 2019; Menezes et al. 2020; Ferreira et al. 2021). For instance, the genus *Acrobrycon* Eigenmann & Pearson, 1924 was initially proposed as most closely related to the genera *Diapoma* Cope, 1894 and *Planaltina* Böhlke, 1954 based on one reproductive character, the presence of pheromone organs overlying the basal portions of the caudal fin, which united the tribe Diapomini (Weitzman et al. 1988). Subsequent morphological studies, examining a large number of osteological and external characters suggested instead that *Acrobrycon* was most closely related to *Mimagoniates* Regan, 1907, *Pseudocorynopoma* Perugia, 1891, and *Diapoma* (Mirande, 2010). More recently, the examination of molecular characters provided additional insights about the relationships of *Acrobrycon*, indicating the genus *Hemibrycon* Günther, 1864 as its sister group (Thomaz et al. 2015). Both *Acrobrycon* and *Hemibrycon* along with *Boehlkea* Géry, 1966 share the presence of teeth along more than one-half the length of the dentigerous margin of the maxilla, which resulted in the reclassification of these three genera in the reclassified tribe Hemibryconini (Thomaz et al. 2015). Further studies based on morphological data recovered *Acrobrycon* as separate from the Hemibryconini, as the sister clade of the Stevardiini (Vanegas-Ríos 2018). Later, based on a combined phylogenetic analysis, Mirande (2019) corroborated a sister-group relationship between *Acrobrycon* and *Hemibrycon*, including both genera within Hemibryconini, a result also shown in Vanegas-Ríos et al. (2020).

Currently, *Acrobrycon* is composed of three species that were revised in Arcila et al. (2013). *Acrobryconipanquianus* (Cope, 1877) is distributed from the western portion of the Amazonas Basin through the north-western region of the La Plata River Basin, including a current junior synonym, *A.tarijae* Fowler, 1940; *A.starnesi* Arcila, Vari & Menezes, 2013, only known from the Thyumayu River in the southwestern portion of the Amazon Basin in Bolivia; and *A.ortii* Arcila, Vari & Menezes, 2013, distributed in the upper Pilcomayo River in Bolivia. Arcila et al. (2013) erroneously indicated the occurrence of *A.ortii* on the northwestern La Plata River Basin including Argentina. However, no references of the presence of this species in this location are listed and the references for valid species from Argentina do not include the occurrence of *A.ortii*, only *A.ipanquianus* as the only confirmed species in this territory (Liotta 2022; Mirande and Koerber 2020).

In 1877, Cope described a wide series of freshwater fish collected by Prof. James Orton (1830–1877) during his exploration of the Upper Amazon in Peru (Cope1877; Fowler 1901) including *Tetragonopterusipanquianus* (Cope, 1877), from the Urubamba River. Following Cope’s description, Fowler (1906) proposed the reclassification of *T.ipanquianus* to *Astyanax* Baird & Girard, 1854. Later, Eigenmann (1910; 1921), reclassified these species to the genus *Hemibrycon*. In 1924, Eigenmann and Person, describing the fishes collected by the biological exploration of Mulford organized by Henry Hurd Rusby (1855–1940), referred to the new specimens from Bolivia at 3080 m.a.s.l., as the same species described by Cope, but this time they proposed a new genus: *Acrobrycon*.

Eigenmann and Pearson (1924) designated *Acrobryconipanquianus* as the type species of the monotypic genus *Acrobrycon*, and indicated a relationship with *Hemibrycon*, with the exception that *Acrobrycon* presented a large caudal pouch observed in mature males. This dimorphism can be clearly seen in the holotype of the species and in the additional materials used to define the genus. In 1940, Fowler described the fishes collected by M. A. Carriker (1879–1965) during 1936 and 1937 in Bolivia, and proposed a new species, *Acrobrycontarijae*. The type locality of this new species was identified as the Lipeo River in Tarija, Bolivia. However, recent studies have shown that Carriker´s route unequivocally puts the collection point of *Acrobrycontarijae* within the Argentinian territory, in the province of Salta (Ringuelet et al. 1967; López et al. 2003; Mirande and Koerber 2020). Fowler differentiated *Acrobrycontarijae* from *A.ipanquianus* (Cope) by the more anterior insertion of the anal fin, which is arranged forward under the front of the base of the dorsal fin, a few more teeth along the maxillary edge, and the dorsal-fin origin clearly closer to the base of the tail than to the tip of the snout. More recently, a morphological revision of the genus *Acrobrycon* conducted by Arcila et al. (2013) using external morphological characters showed that *A.tarijae* could not be distinguished from *A.ipanquianus*, placing *A.tarijae* as a synonym of *A.ipanquianus*. Here, we present new sources of evidence, including osteological and molecular characters from multiple specimens of *Acrobrycon* to evaluate the genetic diversity of the genus and the validity of *A.tarijae* across the La Plata River Basin.

## ﻿Materials and methods

### ﻿Institutional abbreviations

**ANSP**The Academy of Natural Sciences, Drexel University, Philadelphia, Pennsylvania;

**CFA-IC** Colección de Ictiología de la Fundación de Historia Natural Félix de Azara, Buenos Aires, Argentina;

**UNMDP** Instituto de Investigaciones Marinas y Costeras, Universidad Nacional de Mar del Plata, Mar del Plata;

**USNM** National Museum of Natural History, Smithsonian Institution, Department of Vertebrate Zoology, Washington D.C.

Catalog numbers are followed by the total number of samples in alcohol, the number of cleared and stained samples, and the presence of tissue samples from specimens directly preserved in alcohol for molecular studies.

After each collection code, the number of individuals from which measurements (30) were taken or molecularly analyzed (22) is indicated between brackets. Other revised materials were included in the Suppl. material and in the Figs 2, 3.

### ﻿Material examined

**Argentina: SALTA. CFA-IC-4996** [1 with genetic data] Bermejo River and National Route 34 km 1340, near Embarcación. Coll. Y.P. Cardoso, S. Bogan, J.M. Meluso 23°14'58.96"S, 64°8'18.56"W, 10/17/2015; **CFA-IC-5180** [2 with genetic data] Pescado River and National Route 50, near Oran. Coll. Y.P. Cardoso, S. Bogan, J.M. Meluso, (FHN-2293 and 2294), 22°57'53.80"S, 64°21'53.24"W, 10/15/2015; **CFA-IC-5207** [1 with genetic data] Pilcomayo River in Santa María. Coll. Y.P. Cardoso, S. Bogan, J.M. Meluso (FHN-2394), 22°8'7.73"S, 62°48'45.18"W, 10/16/2015; **CFA-IC-5223** [2 with genetic data] Saladillo River and National Route 34 near General Güemes. Coll. S. Bogan, J.M. Meluso (FHN-2120 and 2121), 24°35'42.96"S, 65°4'47.26"W, 10/13/2015; **CFA-IC-5557** [2 with genetic data] Las Conchas River and National Route 9 km 1463, Metan. Coll. J. Montoya-Burgos, Y.P. Cardoso, L.J. Queiroz (AR15-1101 to 1105), 25°28'31.82"S, 64°58'31.46"W, 11/10/2015; **CFA-IC-10369** [3 with morphological data] Las Cañas River, in RP 5, between Lumbrera and Las Víboras (loc. 53). Anta Department. Coll. A. Miquelarena et al., 25°07'S, 64°34'W, 10/11/1988; **CFA-IC-5171** [2 with genetic data] Itiyuro River downstream from the landfill. Coll. Y.P. Cardoso, S. Bogan, J.M. Meluso (FHN-2343 to 2346), 22°6'32.97"S, 63°43'24.44"W, 10/15/2015; **CFA-IC-11453** [8 with morphological data] river in Las Víboras, RP 5, between Las Víboras and Pozo de la Cruz (loc. 54). Anta Department. Coll. R. Menni, A. Miquelarena, 25°00'S, 64°34'W, 10/09/1988; **CFA-IC-11458** [11 with morphological data] first stream after the Juramento River, in Tararipa (loc. 5). Anta Department. Coll. R. Menni and A. Miquelarena, 25°17'S, 64°36'W, 03/28/1987. **UNMDP** [3 with genetic data] Yutón River Route 34. Coll. J.J. Rosso, E. Mabragaña, H. Regidor 23°38'37.73"S, 64°32'23.251"W (UNMDP-4176 to UNMDP-4178), 29/9/2015; [3 with genetic data] Sauzalito River Route 34. Coll. J.J. Rosso, E. Mabragaña, H. Regidor 23°40'16.975"S, 64°33'42.494"W (UNMDP-4198 to UNMDP-4200), 29/9/2015. **SANTIAGO DEL ESTERO. CFA-IC-3165** [2 with morphological data] Horcones River, Locality 13. Coll. Y.P. Cardoso, A. Paracampo, C. Rivera, J. Montoya-Burgos (AR11-939 to 944 and 946 to 950) 26°2'49.68"S, 64°22'8.70"W, 11/27/2011. **TUCUMÁN. CFA-IC-3126** [2 with genetic data – 6 with morphological data] Dulce-Salí River Tributary. Locality 15. Coll. Y.P. Cardoso, A. Paracampo, C. Rivera, J. Montoya-Burgos (AR11-765 to 770) 26°38'01.9"S, 65°03'19.1"W, 11/28 2011; **CFA-IC-5657** [1 with genetic data] Pools linked to the Vipos River. Coll. J. Montoya-Burgos, Y.P. Cardoso, L.J. Queiroz (AR15-1174), 26°29'1.10"S, 65°19'53.40"W, 11/11/2015. **SAN LUIS. CFA-IC-3967** [1 with genetic data] Quinto River and Ruta 14, Justo Daract, Locality 11. Coll. Y.P. Cardoso, A. Jauregüi, M.B. Cabrera (YC13-942), 33°55'7.70"S, 65°9'3.40"W, 11/30/2014. **CÓRDOBA**. [3 with genetic data] Santa Catalina River linked to the Cuarto River. Coll Y.P Cardoso, A. Paracampo, C. Rivera, J. Montoya-Burgos (AR11-1353 to 1355), 33°12'03.2"S, 64°25'43.2"W, 02/12/2011.

### ﻿Molecular approach

**Figure 1.**
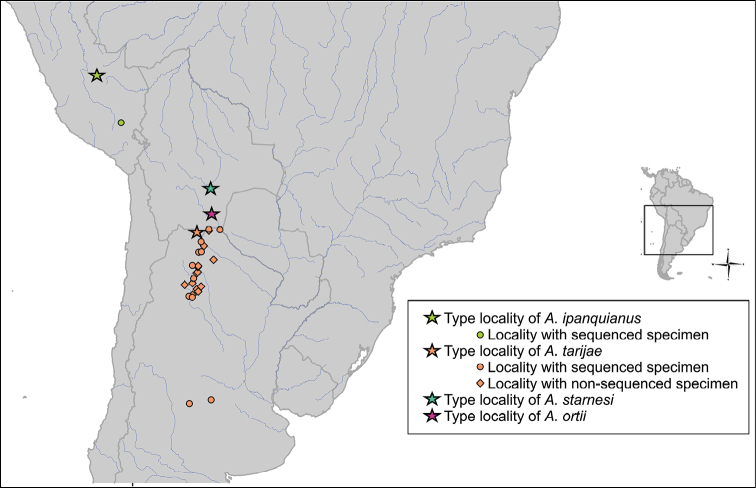
Map of the study area for the species of the genus *Acrobrycon*. Stars represent the type localities for each species; Circles the localities with sequenced specimens; and diamonds the localities without sequenced specimens.

*Phylogenetic reconstruction and haplotype network*. A total of 24 individuals of *Acrobrycon* from 11 localities of La Plata River Basin in Argentina were included in all molecular analyses (Fig. 1). The mitochondrial cytochrome c oxidase I (*COI*) gene was amplified at the Argentine International Barcode of Life Laboratory reference (IIMyC, CONICET, Mar del Plata, Argentina) and the Canadian Centre for DNA Barcoding, Biodiversity Institute of Ontario (CCDB, University of Guelph, Guelph, Ontario) using the cocktail primers described by Ivanova et al. (2007). Sequencing was performed in the CCDB. A molecular phylogenetic approach was used to describe the genetic relationships between the *Acrobrycon* species and some *Hemibrycon* sequences available in GenBank. *Nantisindefessus* (Mirande, Aguilera & Azpelicueta, 2004) and *Diapomaalburnum* (Hensel, 1870) were used as root of the analyses. Maximum likelihood (ML) analyses were conducted using MEGA 7.0.26 (Kumar et al. 2018). Branch support was assessed using the bootstrap algorithm with 1000 replicates. The optimal nucleotide substitution model was selected according to the Bayesian information Criterion (BIC) by JModelTest 2.1.10 (Darriba et al. 2015).

Haplotype network construction is a widely used analysis to assess and visualize the relationships among DNA sequences within a population or species. This approach was effective to explore haplotype partitioning between and within disparate different genetic lineages in a widely distributed Neotropical migratory species (Rosso et al. 2018). Here, this analysis is intended to explore the genetic structure within *A.tarijae* of different basins. The minimum spanning network of the *COI* haplotypes of the *Acrobrycon* sequences was constructed using PopART 1.7 (Leigh and Bryant 2015) to assess the connections and frequencies of haplotypes. The network was colored following the Barcode Index Number (BIN) as indicated below.

**Figure 2.**
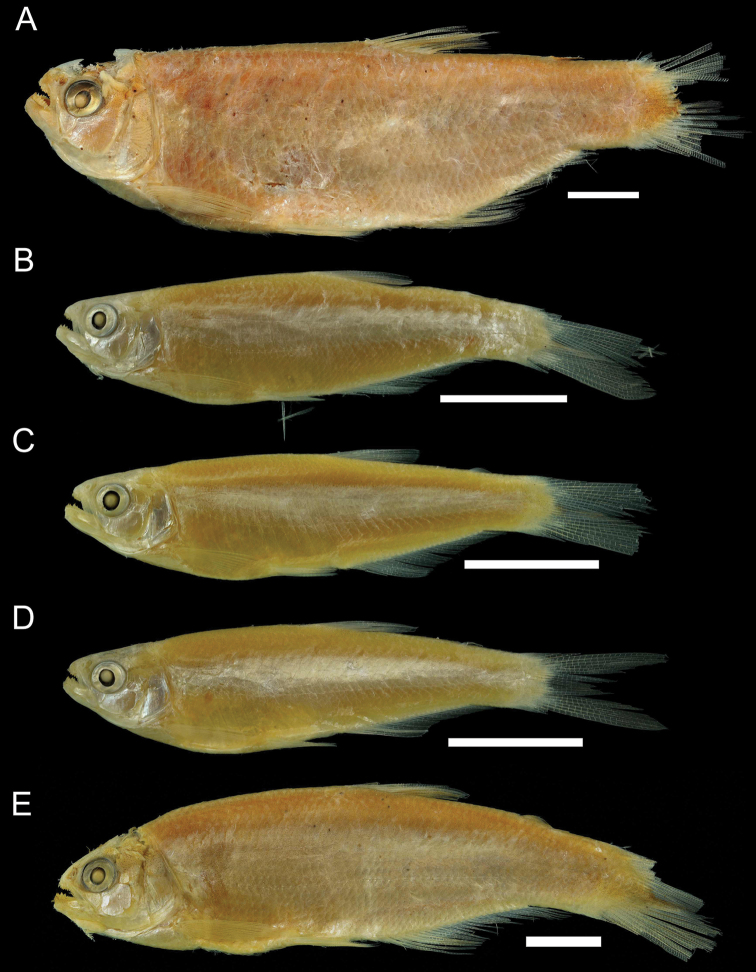
Pictures of the series types of *A.tarijae*. **A** holotype **B–E** paratypes. Scale represents 1 cm. Images from K. Luckenbill, cortesy Academy of Natural Sciences, Drexel University.

Genetic diversity is discussed in the context of three biogeographic processes: isolation-by-distance (IBD; Wright 1943), isolation-by-environment (IBE; Wang and Bradburd 2014) and isolation-by-barrier (IBB; Rahel 2007). To assess the role of geographical distance on the genetic structure of the genus *Acrobrycon*, we used the Mantel test (Mantel 1967) with the aim of testing by the presence of an IBD process. We estimated a matrix of genetic distances using the Kimura two-parameter (K2P) model (Kimura 1980) in MEGA, and a matrix of geographical distances based on individual pair comparisons. To estimate the role of IBB, we classified the sampling localities using two schemes: endorheic basin (no connection to the sea) or exorheic basin (connecting with the sea). The phenomenon of basin fragmentation isolates the aquatic organisms that inhabit these rivers, resulting in an increase of the genetic diversity (Berry et al. 2019). If the time of isolation is long enough, it can result in population genetic differentiation within the species that has been fragmented. But if the time of isolation is even greater, it may eventually lead to allopatric speciation (Dias et al. 2013; Jardim de Queiroz et al. 2017; Briñoccoli et al. 2021). Finally, to assess whether the IBE is structuring the haplotype network of *Acrobrycon* species, we measured the altitude in meters above sea level for each site in which specimens of *Acrobrycon* were collected.

**Figure 3.**
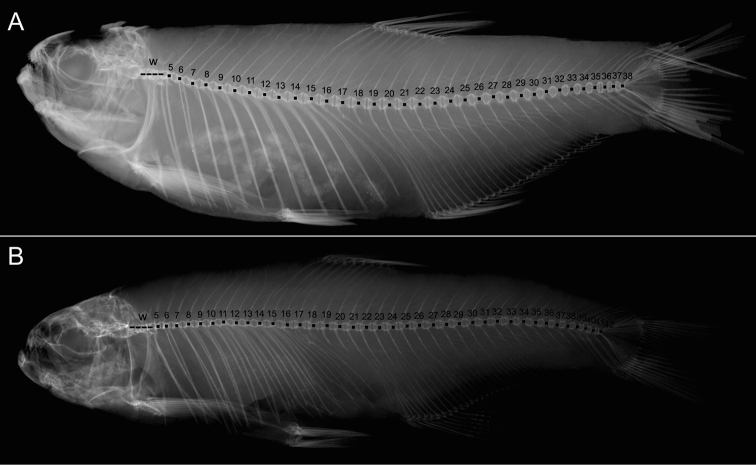
Count of vertebrae in holotypes of **A***A.tarijae* and **B***T.ipanquianus*. Abbreviation W represents the first four vertebrae of the Weber complex. Images from K. Luckenbill, cortesy Academy of Natural Sciences, Drexel University.

*Diversity and divergence*. Species delimitation analyses were conducted using the Automatic Barcode Gap Discovery (ABGD) method (Puillandre et al. 2012) and the BIN. For the ABGD, the *COI* alignment was uploaded to the online platform (http://wwwabi.snv.jussieu.fr/public/abgd/abgdweb.html) and run using two schemes: the default settings (Pmin = 0.001, Pmax = 0.1, Steps = 10, X relative gap width = 1.5, Nb bins = 20), and the Kimura distance models. The BIN was generated and downloaded for all 22 sequences available on the BOLD database. The BIN analysis clusters barcode sequences to create Operational Taxonomic Units (OTUs) that closely reflect species groupings. We computed the distance matrix of the K2P model (Kimura, 1980) using MEGA following the groups identified in the ABGD and BIN analyses. Finally, we explored whether the groups identified by the BIN and ABGD analyses were consistently supported by the ML analysis and the morphological characters (Tables 1, 2).

**Table 1.** Morphometric measurements of 30 specimens of *Acrobrycontarijae*. Standard length (SL) is expressed in mm and all other measurements are expressed as a percentage of SL, except for head subunits which are expressed as percentages of the head length.

	Range	Mean ± S.D.
Standard length (mm)	40.5–90	62.12±12.89
**percent of SL**		
Depth at dorsal-fin origin	11.8–30.5	16.99±5.21
Snout to dorsal-fin origin	22.8–47.8	33.4±6.71
Snout to pectoral-fin origin	10.5–20.9	15.16±2.71
Snout to pelvic-fin origin	20.6–45.3	28.81±6.66
Snout to anal-fin origin	26.4–59.7	39.05±8.92
Caudal peduncle depth	5–12	7.76±1.73
Caudal peduncle length	4.4–13	7.75±2.29
Pectoral-fin length	9–18.6	13.05±2.51
Pelvic-fin length	5.6–12.8	9.17±1.76
Dorsal-fin base length	4.2–11	7.45±2.02
Dorsal-fin height	8.6–18.3	13.14±2.57
Anal-fin base length	10–27.6	17.82±3.86
Anal-fin lobe length	12.8–33.7	21.91±4.63
Eye to dorsal-fin origin	18.3–40	26.99±5.81
Dorsal-fin origin to caudal-fin base	18.5–45.5	31.39±6.98
Head length	9.8–19.2	14.11±2.71
**percent of HL**		
Horizontal eye diameter	3–5.7	4.42±0.67
Least interorbital width	3–6.3	4.47±0.89

**Table 2.** Meristic data taken in 30 specimens of *A.tarijae*.*Data taken in 11 specimens.

Lateral line scales	51–60
Dorsal-fin rays	ii,8
Anal-fin rays	v-vi, 23–27
Pelvic-fin rays	i,6–7
Pectoral-fin rays	i,9–11
Maxillary teeth	6–11
*Vertebrae	37–39
*Pleural ribs	12–13

### ﻿Morphological data remarks

Measurements rounded to the nearest 0.1 mm were made with digital calipers. Counts and measurements were conducted following previously standardized protocols (Fink and Weitzman 1974; Menezes and Weitzman 1990, 2009; Menezes et al. 2003). In order to standardize as much as possible our comparisons with previous studies examining all species in the genus *Acrobrycon*, measurements and counts were directly compared to those obtained by Arcila et al. (2013).

Meristic counts of 30 specimens are provided in the species description (See Tables 1, 2). Vertebral counts were taken from cleared and stained specimens (CFA-IC-10058; Suppl. material 1: Fig. S3) prepared following the proposed procedures by Taylor and Van Dyke (1985) and dried skeletons (CFA-IC-9504 and CFA-IC-9505) (*N* = 5) and X-ray images of six specimens of the type series of *A.tarijae* (Holotype: ANSP 68775, paratypes ANSP 68776, and ANSP 68778). Vertebral counts of *A.ipanquianus* were performed from the holotype, ANSP 21114, and two paratypes, ANSP 21115. The number of vertebrae of the Weberian apparatus was quantified as four elements, and the first pre-ural center and the first fused ural of the caudal fin were counted as a single element.

## ﻿Results

### ﻿Molecular

*Phylogenetic reconstruction and haplotype network*. A total of 22 sequences from the original 24 specimens of *A.tarijae* processed were obtained and deposited in GenBank (accession numbers: MW940261-MW940282). *COI* sequences were also uploaded to BOLD under the project “COIPE Peces Argentinos”. Sequences of two specimens of *A.ipanquianus* from the Amazon Basin, Peru and 18 species from the genus *Hemibrycon* were obtained from GenBank for downstream phylogenetic comparisons. The Tamura-Nei model+G+I (TN93+G+I) was chosen as the best nucleotide substitution model under the BIC.

**Figure 4.**
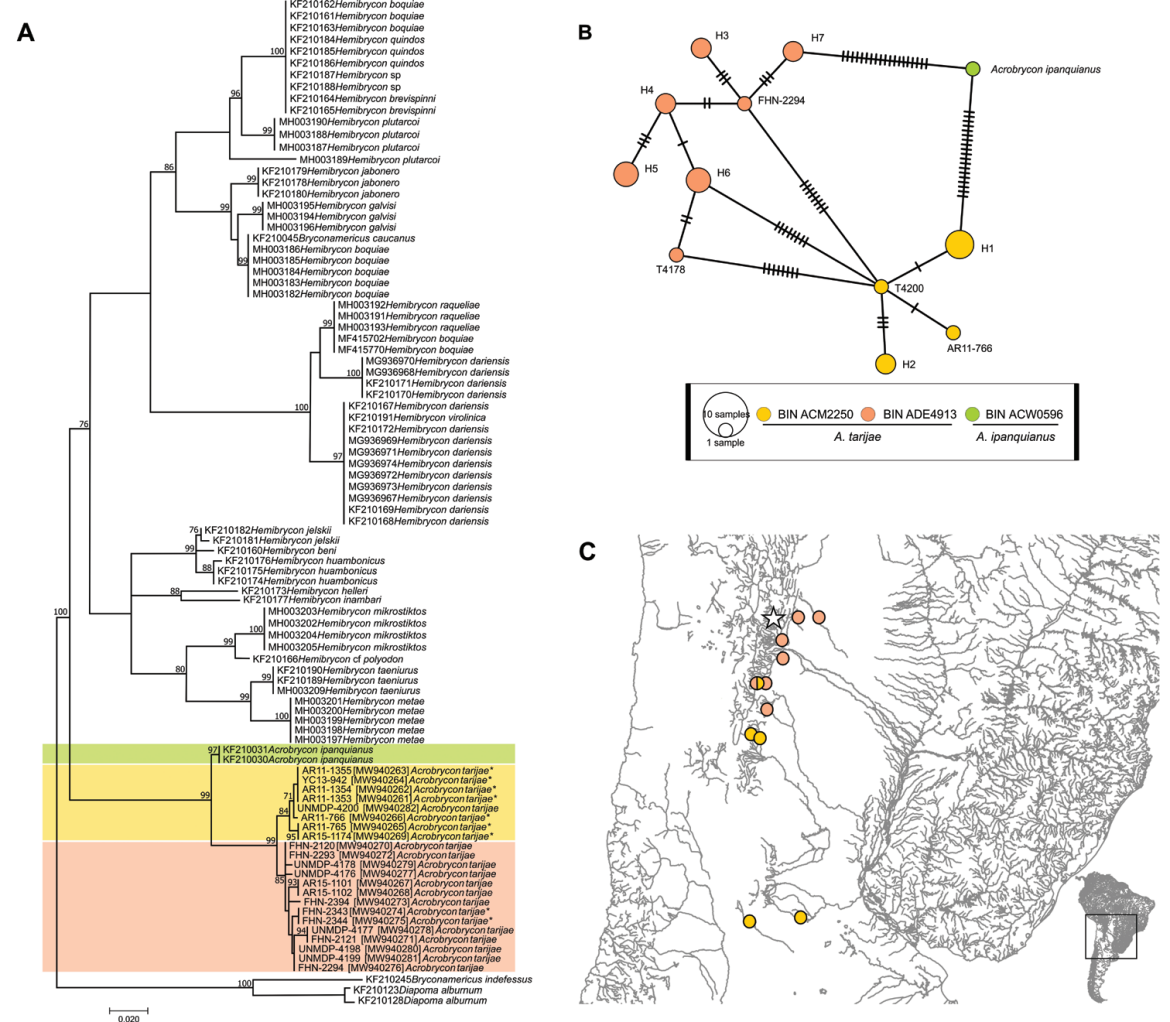
**A** Maximum likelihood tree of *Acrobrycon* based on 521 nucleotides of the mitochondrial gene *COI*. Bootstrap values are shown above the branches, values below 70 are not shown. Genbank access numbers are indicated for *A.tarijae*, and an asterisk indicates those that were sampled in endorheic basins **B** haplotype network, colored by three BINs groups **C** map with the sampling sites of the *A.tarijae* specimens.

Our phylogenetic results supported the sister-group relationships between the species under study of the genera *Acrobrycon* and *Hemibrycon* with strong bootstrap support (Fig. 4A). The relationships within *Acrobrycon* under study resulted in two major clades, the first one including only the specimens of *A.ipanquianus*, and the second clade encompassing the 22 specimens of *A.tarijae* from La Plata River Basin.

We found a total of 11 different haplotypes in *A.tarijae* from La Plata Basin in Argentina, and one haplotype in *A.ipanquianus* from Peru (Fig. 4B). The haplotype network provided strong support differentiating lineages *A.ipanquianus* from *A.tarijae*. Interestingly, it also identified two haplotype groups among the specimens of *A.tarijae* that differ in their assigned BIN and in their distinctive geographic distribution (Fig. 4C). This result is consistent with the inferred phylogenetic tree (Fig. 4A), and the Mantel test that showed a significant correlation between geographic and genetic distances among the *A.tarijae* specimens (*p* = 0.008), indicating possibly an IBD process.

Regarding the type of basin (IBB), the sites located in the north of Argentina (Salta and Jujuy) are part of exorheic basins, while those sampling sites towards the centre-south of the country (Tucumán, Córdoba and San Luis) are part of endorheic-arheic basins (Fig. 4A). We also found the two groups of haplotypes separated from each other by eight mutations in the haplotype network colored according to BIN values (Fig. 4B). In terms of environment (IBE), altitudes in sampling sites spanned from 182 to 883 MASL.

*Diversity and divergence*. Three BINs were obtained for the genus *Acrobrycon*. The ACW0596 barcode was assigned to two specimens of *A.ipanquianus* from Peru. We found two barcodes (ACM2250, ADE4913) associated to *A.tarijae* in Argentina. The barcode ACM2250 was assigned to seven specimens from Salí, Bermejo, Cuarto and Quinto rivers, southern and northern drainages in Argentina, while the barcode ADE4913 was found on nine individuals from Bermejo and Pilcomayo rivers, northern drainages from the same country. However, the ABGD analysis only reported two groups, one corresponding to the ACW0596BIN and another corresponding to both ACM2250 and ADE4913 BINs together. The within-BIN distances were zero for the ACW0596 and 0.01% for both ACM2250 and for ADE4913. The between-BIN distances were 1.87% ACM2250–ADE4913; 4.2% ACW0596–ACM2250 and 4.1% for ACW0596–ADE4913.

### ﻿Morphology

#### ﻿ Acrobrycon

Taxon classificationAnimaliaCharaciformesCharacidae

Eigenmann & Pearson, 1924

825B6CDB-DBF0-5D86-809C-E729A2DAD8BB

##### Type species.

*Tetragonopterusipanquianus* Cope, 1877, by original description (Suppl. material 1: Fig. S1).

#### ﻿ Acrobrycon tarijae

Taxon classificationAnimaliaCharaciformesCharacidae

Fowler, 1940

2DE9E4FC-344F-5A1D-9BDC-D0C443012580

Figs 2 , 3 , Tables 1 , 2 , Suppl. material 1: Fig. S2

Acrobrycon tarijae Fowler, 1940:50 [Type locality: Lipeo River, branch of Bermejo River, Department of Tarija, Bolivia (currently the Lipeo River type locality was relocated to the province of Salta, Argentina)].

##### Amended diagnosis.

*Acrobrycontarijae* is distinguished from *A.ipanquianus* by the lower number of vertebrae [37 (1), 38 (3+holotype+3 paratypes), 39 (1+2 paratypes) vs. 41 (2 paratypes), 42 (holotype+2 paratypes)], and pleural ribs (12 (1 paratype), 13 (5+holotype+4paratypes) pairs vs. 14 (holotype+2 paratypes) pairs in the type series). *Acrobrycontarijae* is distinguished from *A.starnesi* by the number of perforated scales of the lateral line [51 (1), 52 (1), 54 (4), 55 (2), 56 (1) 57 (7), 58 (5), 59 (7), 60 (2) vs. 61 to 66], and the number of horizontal-scale rows around the caudal peduncle (19 (12), 20 (9), 21 (9) vs. 22 to 26). *Acrobrycontarijae* can be distinguished from *A.ortii* by the number of branched anal-fin rays [23 (3), 24 (9), 25 (10), 26 (7), 27 (1) vs. 19 to 21].

##### Description.

The description of *A.tarijae* follows Arcila et al. (2013), including some adjustments to the ranges as indicated below. *Acrobrycontarijae* is a characid of moderate size (can exceed 114 mm of standard length, SL), with an elongated body. Greater body depth in the sector behind the origin of the pectoral fins and before the dorsal-fin origin. Dorsal profile convex from the tip of the snout to the origin of the dorsal fin, slightly depressed along the nape, almost straight along the base of the dorsal fin and slightly concave along the caudal peduncle. Ventral profile convex. Dorsal-fin origin anteriorly than the origin of the anal fin. Rounded muzzle in lateral view.

Terminal mouth slightly upwards. Maxilla extending posteriorly beyond the vertical through the anterior margin of the orbit, but not reaching the vertical through the posterior border of the pupil. Posteroventral border of the maxilla convex and posterior margin concave. Premaxillary teeth in two different rows, outside row with 4–5 teeth and internal row with 4 teeth. Larger teeth with five cusps; smaller teeth with three cusps. Maxillary teeth 6–11. Larger anterior maxillary teeth with 1–3 cusps, other smaller teeth with 1–2 cusps. Dentary with 4 large anterior teeth with 5 cusps, followed by 6–10 smaller teeth with 1–3 cusps.

Tip of the pelvic fin does not reach the anal-fin origin, cycloid scales, with 4 to 8 rays along the exposed surface over most of the body and 14 to 17 rays on scales bordering the opening of the caudal pocket. Lateral line with 51–60 perforated scales. Predorsal scales 19–26. Horizontal-scale rows around caudal peduncle 19–21.

Dorsal-fin rays ii+8. Some specimens with posteriormost dorsal ray unbranched and others with a branched condition in this ray, although this is restricted to a very small part of its distal tip. Small adipose fin. Anal fin with v,vi-23–26. Pectoral fin with i, 9–10. Pelvic-fin rays i, 7 (12), 8 (18). Caudal fin with two well differentiated lobes, with i, 9–10.

##### Sexual dimorphism.

Mature males present a hypertrophied terminal caudal-fin squamation forming a caudal pocket and also have bony hooks on the anal, pelvic, and caudal-fin rays.

##### Coloration in live specimens.

Gray ochre dorsal coloration from the nostrils to the caudal peduncle. Silvery flanks, paler at ventral section and with purplish-bluish reflection dorsally. A golden to greenish coloration bordering the lateral line. A silver wide band behind the humeral spot progressing distally to the end of the body, continuing with a marked black pigmentation in the medial rays of the caudal fin. Circum-orbital bones generally silver, the fifth and sixth infraorbitals may have purplish reflections. Operculum with conspicuous violet reflections, turning greenish above and ahead this bone. A conspicuous humeral dark spot vertically elongated. Pectoral and ventral fins whitish. The dorsal and adipose fins smoothly yellowish. The anal fin gray or yellowish usually with a whitish lower margin (Suppl. material 1: Fig. S2).

##### Coloration in alcohol.

Preserved specimens, body brown, with darker dorsum. Humeral spot dark, with a well-defined upper part and a fainter lower expansion. In many specimens the lower expansion of humeral spot absent. The lateral band dark, thin at the level of the humeral spot, and shortly wider until the distal end of the body. This band conspicuous in some specimens and very faint in others. The middle rays of the caudal fin black.

## ﻿Discussion

### ﻿Species diversity in *Acrobrycon*

Our study revealed a greater genetic diversity than expected for the genus *Acrobrycon* along La Plata River Basin as well as additional taxonomically informative morphological characters allowing the discrimination between two of its previously synonymized species. Particularly, our results from an integrative analytical approach allows us to revalidate *A.tarijae*, which is distinguished from *A.ipanquianus* by the lower number of vertebrae and pleural ribs and a higher number of unbranched anal-fin rays. The genetic analyses, despite involving only one marker, showed two clear clades, one with samples identified as *A.ipanquianus* and the other as *A.tarijae*. The morphological traits of the specimens of *A.tarijae* showed clear differences from the two other species described for this genus, *A.ortii* and *A.starnesi* (Arcila et al. 2013) in the branched anal-fin rays and scales. Furthermore, the molecular analyses showed a conspicuous genetic structure within *A.tarijae* revealing the existence of two mitochondrial lineages. The BIN analyses assigned two different OTUs to these lineages which diverged by 1.87%. In fishes, *COI* sequences have traditionally been used to delimit species and a 2% paired divergence threshold has been proposed to discriminate interspecific from intraspecific genetic divergence (Ward et al. 2009; Ward 2012). However, the use of only this genetic criterion for taxonomic decision has been controversial. In *Hypostomus*, for example, it was shown that 82% of the sister-species pairs with well-defined morphology, showed genetic divergence values of less than 2% (Cardoso et al. 2019; Jardim de Queiroz et al. 2020; Cardoso et al. 2021). Despite our genetic results obtaining a distance close to 2% of differentiation, our examination of morphological characters did not find unequivocal characters for the description of a new species.

### ﻿Genetic structure within *Acrobrycontarijae*

Our results indicated that the distribution of *A.tarijae* is restricted to the western headwaters of La Plata Basin (Liotta 2022), including an important sector of the Chaco plain on the main channel of the Pilcomayo River and the dry Chaco sector of the Bermejo Basin in Formosa (Ringuelet et al. 1967). The geographic coverage of our samplings extends the distribution of *A.tarijae* to the Quinto River basin in San Luis, being the only record known for the province of San Luis and the southernmost documented report for this species (Cardoso et al. 2015). However, the abundance of this species is relatively low compared to the populations distributed in the Andean foothills or the Pampas mountains, also linked to landscapes of the foothills (Monasterio de Gonzo 2003; Mirande and Aguilera 2009). *Acrobrycontarijae* is an abundant species in some endorheic basins such as the Itiyuro, the Horcones and the Urueña rivers (Monasterio de Gonzo 2003; Monasterio de Gonzo et al. 2006), and in the large arheic system of Mar Chiquita Lagoon Basin (Haro et al. 1991; Butí and Miquelarena 1995; Haro and Bistoni 1996). These endorheic basins are characterized by high diversity of endemic freshwater fishes (Miquelarena and Menni 1999). Briñoccoli et al. (2021) observed that the type (endorheic vs. exorheic) of basin played a predominant role in population structure (as well as the altitude at which the rivers were located) in *Jenynsialineata*, a small ovoviviparous fish widely distributed throughout the La Plata Basin. As well as in *A.tarijae*, Briñoccoli et al. (2018) showed that the catfish *Rhamdellaaymarae*, originally described from an endorheic basin, has a wider distribution.

### ﻿Morphological variations in *Acrobrycon*

The morphological study by Arcila et al. (2013) characterized *A.ipanquianus* (including *A.tarijae* as its synonym) as a species with a relatively deep body, a trait that would significantly differentiate it from *A.ortii*. However, in the type series of this species (holotype and more than 33 paratypes) some individuals (see for instance Fig. 2 B–D) have a low body with proportions similar to those described for *A.ortii*. According to Arcila et al. (2013) the explanation for the lack of variation within the *Acrobrycon* species may be associated with the relatively homogeneous habitats occupied by these species and their moderate-sized geographic distributions. However, in the La Plata River Basin, a great morphological variation of body depth was observed in *A.tarijae*. No link was found between observed genetic differences and these morphological variations. In our survey, elongated, low and well-stylized bodies are observed in individuals that inhabit rivers with strong currents, sometimes linked to mountain formations or in the beds of large rivers, such as the Pilcomayo River. Morphological differences between populations of the same species could be due to the environmental conditions to which the fish are exposed. Due to the evident lack of diagnostic value of body depth to discriminate *Acrobrycon* species, the number of branched rays in the anal fin remains an important diagnostic character to differentiate *A.ortii* from other species (19–21 vs. 23–27 in *A.ipanquianus* and *A.tarijae*). Unfortunately, the molecular identity of *A.ortii* and *A.starnesi* could not be assessed in this study, but our results urge the inclusion of these species along with additional populations and markers of *Acrobrycon* in future studies to investigate their population structures and phylogenetic relationships as well as biogeographic and morphological patterns of diversification.

## Supplementary Material

XML Treatment for Acrobrycon

XML Treatment for Acrobrycon tarijae

## Supplementary materials

Supplementary material 1 Molecular and taxonomic evidence unmask hidden species diversity in the genus *Acrobrycon* (Characiformes, Characidae). Fig. S1–S3. Data type material, images Explanation note List of all Material examined, types of *T.ipanquianus*, living specimen of *Acrobrycontarijae* and vertebrae count of two diaphanous specimens of *A.tarijae*. **Fig. S1.** Types of *T.ipanquianus*. **A, B** paratypes **C** holotype. The scale represents 1 cm. **Fig. S2.** Living specimen of *Acrobrycontarijae*, **A** from Las Conchas River, Salta Argentina (CFA-IC-5557) **B**, **C** from Rosario River, Salta Argentina (CFA-IC-5017). **Fig. S3.** Vertebrae count of two diaphanous specimens of *A.tarijae* (CFA-IC-10058), Bermejo River basin, Argentina. The abbreviation W represents the first four vertebrae of the Weber complex. Scale bar: 1 cm. https://binary.pensoft.net/file/666843This dataset is made available under the Open Database License (http://opendatacommons.org/licenses/odbl/1.0/). The Open Database License (ODbL) is a license agreement intended to allow users to freely share, modify, and use this Dataset while maintaining this same freedom for others, provided that the original source and author(s) are credited.Yanina F. Briñoccoli, Sergio Bogan, Dahiana Arcila, Juan J. Rosso, Ezequiel Mabragaña, Sergio M. Delpiani, Juan Martín Díaz de Astarloa, Yamila P. Cardoso
