# Supplementary material for: ﻿Molecular and morphological evidence revalidates Acrobrycontarijae (Characiformes, Characidae) and shows hidden diversity
Source: Zookeys. 2022 Mar 31;1091:99–117. doi: 10.3897/zookeys.1091.73446 (PMC9005463; doi:10.3897/zookeys.1091.73446)

Molecular and taxonomic evidence unmask hidden species diversity in the genus *Acrobrycon* (Characiformes: Characidae)

Yanina F. Briñoccoli, Sergio Bogan, Dahiana Arcila, Juan J. Rosso, Ezequiel Mabragaña, Sergio M. Delpiani, Juan Martín Díaz de Astarloa, Yamila P. Cardoso

Supporting Information 2

List of all Material examined

Institutional abbreviations are: ANSP: The Academy of Natural Sciences, Drexel University, Philadelphia, Pennsylvania. CFA-IC: Colección de Ictiología de la Fundación de Historia Natural Félix de Azara, Buenos Aires, Argentina. UNMDP: Instituto de Investigaciones Marinas y Costeras, Universidad Nacional de Mar del Plata, Mar del Plata.USNM: National Museum of Natural History, Smithsonian Institution, Department of Vertebrate Zoology, Washington D.C. Catalog numbers are followed by the total number of samples in alcohol, the number of c & s samples, and the presence of tissue samples from specimens directly preserved in alcohol for molecular studies (FHN, AR, or YC).

Argentina: SALTA. CFA-IC-1635 (1) El Rey River, Bermejo Basin, Coll. D. Rumiz, 07/14/1979; **CFA-IC-1639** (14) Dorado River, Depto. Anta. Coll. Pierotti and Boudin, 12/26/1955; **CFA-IC-1653** (3) Urueña River, border between Salta and Tucuman and Route 34; Coll. Gómez, Quintana, 03/12/1992; **CFA-IC-3351** (12) El Rey National Park. Coll. A. Miquelarena, 07/14/1979; **CFA-IC-4996** (1) Bermejo River and National Route 34 km. 1340, near Embarcación. Coll. Y.P. Cardoso, S. Bogan, J.M. Meluso (FHN-2623) 23°14'58.96" S / 64°8'18.56" W, 10/17/2015. **CFA-IC-5017** (1) Rosario River near Ovando. Coll. S. Bogan, J.M. Meluso (FHN-1736), 25°48'10.82" S / 65°4'38.90" W, 10/10/2015; **CFA-IC-5058 (5)** Las Conchas River and National Route 68 km. 26, near Las Conchas. Coll. S. Bogan, J.M. Meluso (FHN-1870 to 1874) 25°58'28.89" S / 65°46'5.66" W, S. Bogan, 10/11/2015; **CFA-IC-5067** (3) Las Conchas River and National Route 68 km. 38, near Santa Bárbara, Coll. S. Bogan, J.M. Meluso (FHN-1850 to 1852), 25°55'10.71" S / 65°42'56.67" W, 10/11/2015; **CFA-IC-5171** (4) Itiyuro River downstream from the landfill. Coll. Y.P. Cardoso, S. Bogan, J.M. Meluso (FHN-2343 to 2346), 22°6'32.97" S / 63°43'24.44" W, 10/15/2015; **CFA-IC-5180** (2) Pescado River and National Route 50, near Oran. Coll. Y.P. Cardoso, S. Bogan, J.M. Meluso, (FHN-2293 and 2294), 22°57'53.80" S / 64°21'53.24" W, 10/15/2015; **CFA-IC-5207** (1) Pilcomayo River in Santa María. Coll. Y.P. Cardoso, S. Bogan, J.M. Meluso (FHN-2394), 22°8'7.73" S / 62°48'45.18" W, 10/16/2015; **CFA-IC-5223** (2) Saladillo River and National Route 34 near General Güemes. Coll. S. Bogan, J.M. Meluso (FHN-2120 and 2121), 24°35'42.96" S / 65°4'47.26" W, 10/13/2015; **CFA-IC-5349** (1) river in Las Víboras, Provincial

Route 5, between Las Víboras and Pozo de la Cruz. Coll. R.C. Menni, A. Miquelarena, J. Casciotta, 08/10/1988; **CFA-IC-5464** (1) Capiazuti River. Loc. 56. Coll. R.C. Menni, A. Miquelarena, J. Casciotta, October 1988; **CFA-IC-5557** (5) Las Conchas River and National Route 9 km. 1463, Metan. Coll. J. Montoya-Burgos, Y.P. Cardoso, L.J. Queiroz (AR15-1101 to 1105), 25°28'31.82" S / 64°58'31.46" W, 11/10/2015**; CFA-IC-10058** (11) El Rey National Park. Anta Department. Coll. D. Rumiz, 58°24'42" S / 64°38' W, 07/14/1979; **CFA-IC-10062** (3) El Rey National Park. Anta Department. Coll. D. Rumiz, 62°24'42" S / 64°38' W, 01/14/1980; **CFA-IC-10368** (3) Ovejería Stream, El Rey National Park. Anta Department. Coll. D. Rumiz, 24°38' S / 64°36' W, 04/14/1980; **CFA-IC-10369** (14) Las Cañas River, in RP 5, between Lumbrera and Las Víboras (loc. 53). Anta Department. Coll. A. Miquelarena et al., 25°07' S / 64°34' W, 10/11/1988; **CFA-IC-11453** (109) river in Las Víboras, RP 5, between Las Víboras and Pozo de la Cruz (loc. 54). Anta Department. Coll. R. Menni, A. Miquelarena, 25°00' S / 64°34' W, 10/09/1988; **CFA-IC-11458** (24) first stream after the Juramento River, in Tararipa (loc. 5). Anta Department. Coll. R. Menni and A. Miquelarena, 25°17' S / 64°36' W, 03/28/1987; **CFA-IC-11465** (1) first stream after the Juramento River, in Tararipa (loc. 5). Anta Department. Coll. R. Menni and A. Miquelarena, 25°17' S / 64°36' W, 03/28/1987; **CFA-IC-11476** (82) Metán River on RP 46, road between Punta de Agua and La Costosa (loc. 2). Metán Department. Coll. R. Menni, A. Miquelarena, 25°31' S / 64°38' W, 03/28/1987; **CFA-IC-11505** (24) Las Cañas River, on RP 5, between Lumbrera and Las Víboras (loc. 53). Anta Department. Coll. R. Menni, A. Miquelarena, 25°07' S / 64°34' W, 10/09/1988. **JUJUY. CFA-IC-10340** (4) Aguas Calientes Stream. Santa Barbara Department. Coll. R. Menni, A. Miquelarena, J. Casciotta, 23°44' S / 64°38' W, 03/29/1987; **CFA-IC-11501** (5) Zanjón Seco Stream, N of Libertador General San Martín, RN 34 (loc. 64). Ledesma Department. Coll. R. Menni, H. López and S. Gómez, 23°41' S / 64°34' W, 08/17/1991. **UNMDP** (3) Yutón River Route 34. Coll. J.J. Rosso, E. Mabragaña, H. Regidor 23°38'37.73" S / 64°32'23.251" W (UNMDP-4176 to UNMDP-4178), 29/9/2015; (3) Sauzalito River Route 34. Coll. J.J. Rosso, E. Mabragaña, H. Regidor 23°40'16.975" S / 64°33' 42.494" W (UNMDP-4198 to UNMDP-4200), 29/9/2015. **FORMOSA. CFA-IC-8736** (2) Santa Teresa, Pilcomayo River. Coll. A. Paracampo, D. Barrasso, 22º42'7,4" S / 62º12'31,7" W, 08/24/2006. **SANTIAGO DEL ESTERO. CFA-IC-3165** (11) Horcones River, Locality 13. Coll. Y.P. Cardoso, A. Paracampo, C. Rivera, J. Montoya-Burgos (AR11-939 to 944 and 946 to 950) 26°02, 828' S / 64°22,145' W, 11/27/2011; **CFA-IC-9504** (1) Horcones River, Locality 13. Coll. Y.P. Cardoso, A. Paracampo, C. Rivera, J. Montoya-Burgos (AR11-945), 26°02, 828' S / 64°22,145' W, 11/27/2011. **TUCUMAN. CFA-IC-3126** (6) Dulce-Salí River Tributary. Locality 15. Coll. Y.P. Cardoso, A. Paracampo, C. Rivera, J. Montoya-Burgos (AR11-765 a 770) 26°38'01.9" S / 65°03'19.1" W, 11/28 2011; **CFA-IC-10076** (2) Calera River. Burruyacú Department. Coll. R. Menni, H. López, J. Casciotta, 26°37' S / 65°04' W, 05/11/1980;

CFA-IC-10082 (7) Chuscha River, La Higuera. Trancas Department. Coll. C. Butí and Lozano, 26°23' S / 64°36' W April 1987; **CFA-IC-5657** (1) Pools linked to the Vipos River. Coll. J. Montoya-Burgos, Y.P. Cardoso, L.J. Queiroz (AR15-1174), 26°29'1.10" S / 65° 19'53.40" W, 11/11/2015. **CORDOBA. CFA-IC-9505** (1) La Carlota, Cuarto River. Coll. Y.P. Cardoso (YC09-009), 33°24'30.6"S / 63°17'46.4"W, 08/14/2009. **SAN LUIS. CFA-IC-3967** (1) Quinto River and Ruta 14, Justo Daract, Locality 11. Coll. Y.P. Cardoso, A. Jauregüi, M.B. Cabrera (YC13-942), 33°55'7.70" S / 65°9'3.40" W, 11/30/2014.

Figure S1. Types of *T. ipanquianus*. A-B: Paratypes and C: Holotype. The scale represents 1 cm.

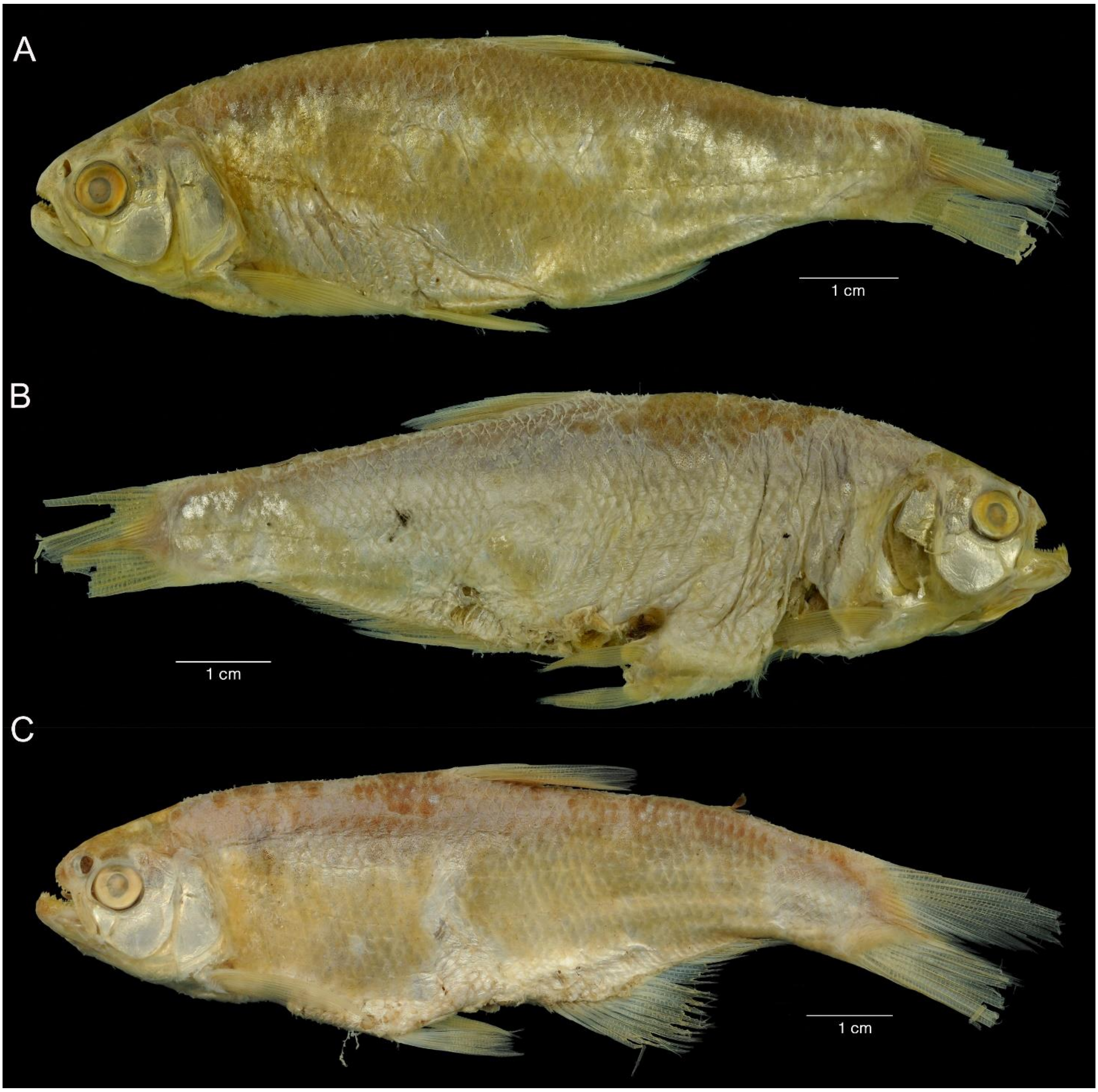

Figure S2. Living specimen of *Acrobrycon tarijae*, A from Las Conchas River, Salta Argentina (CFA-IC-5557); B and C from Rosario River, Salta Argentina (CFA-IC-5017).

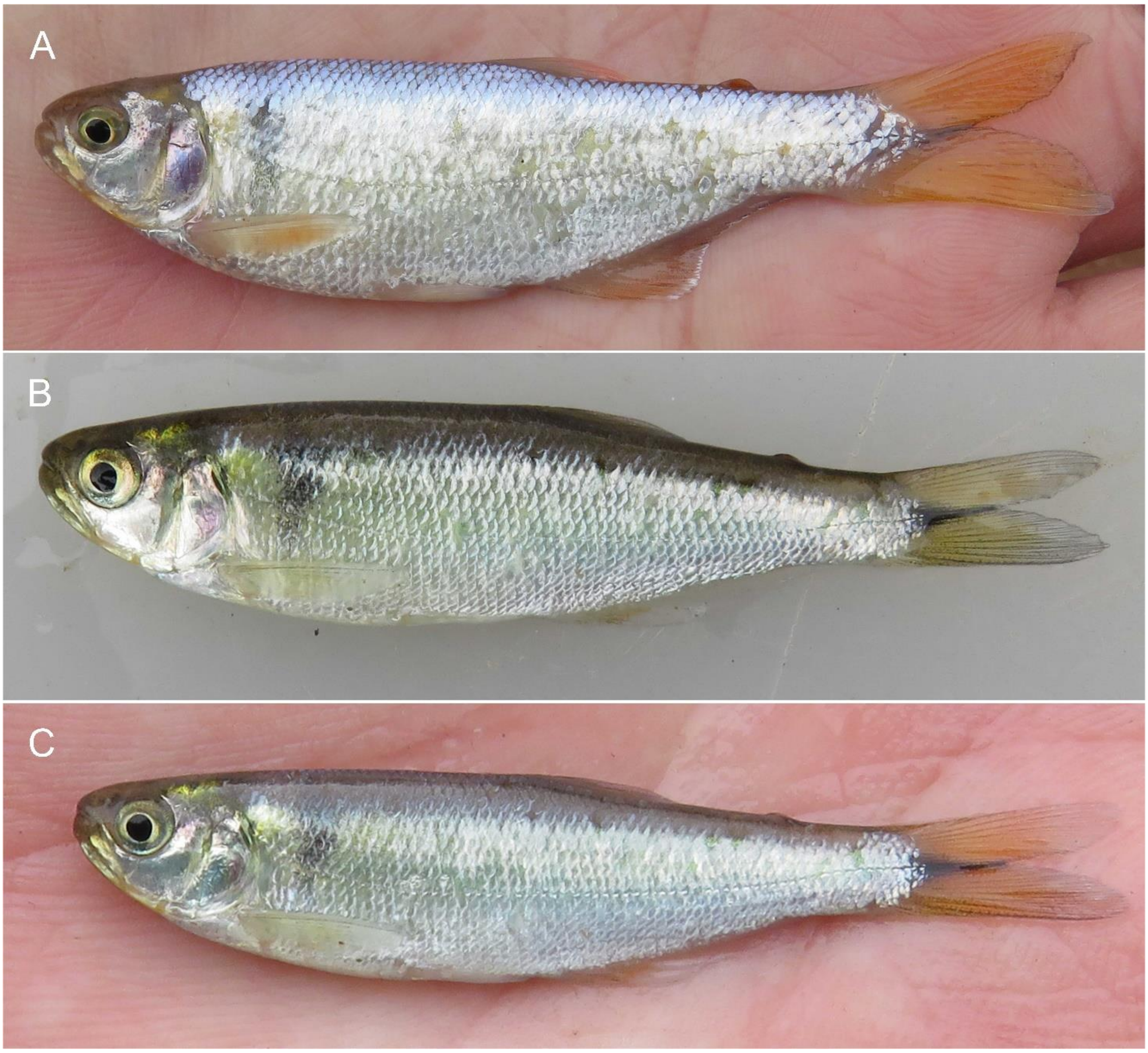

Figure S3. Vertebrae count of two diaphanous specimens of *A. tarijae* (CFA-IC-10058), Bermejo river basin, Argentina. The abbreviation W represents the first four vertebrae of the Weber complex. The scale represents 1 cm.

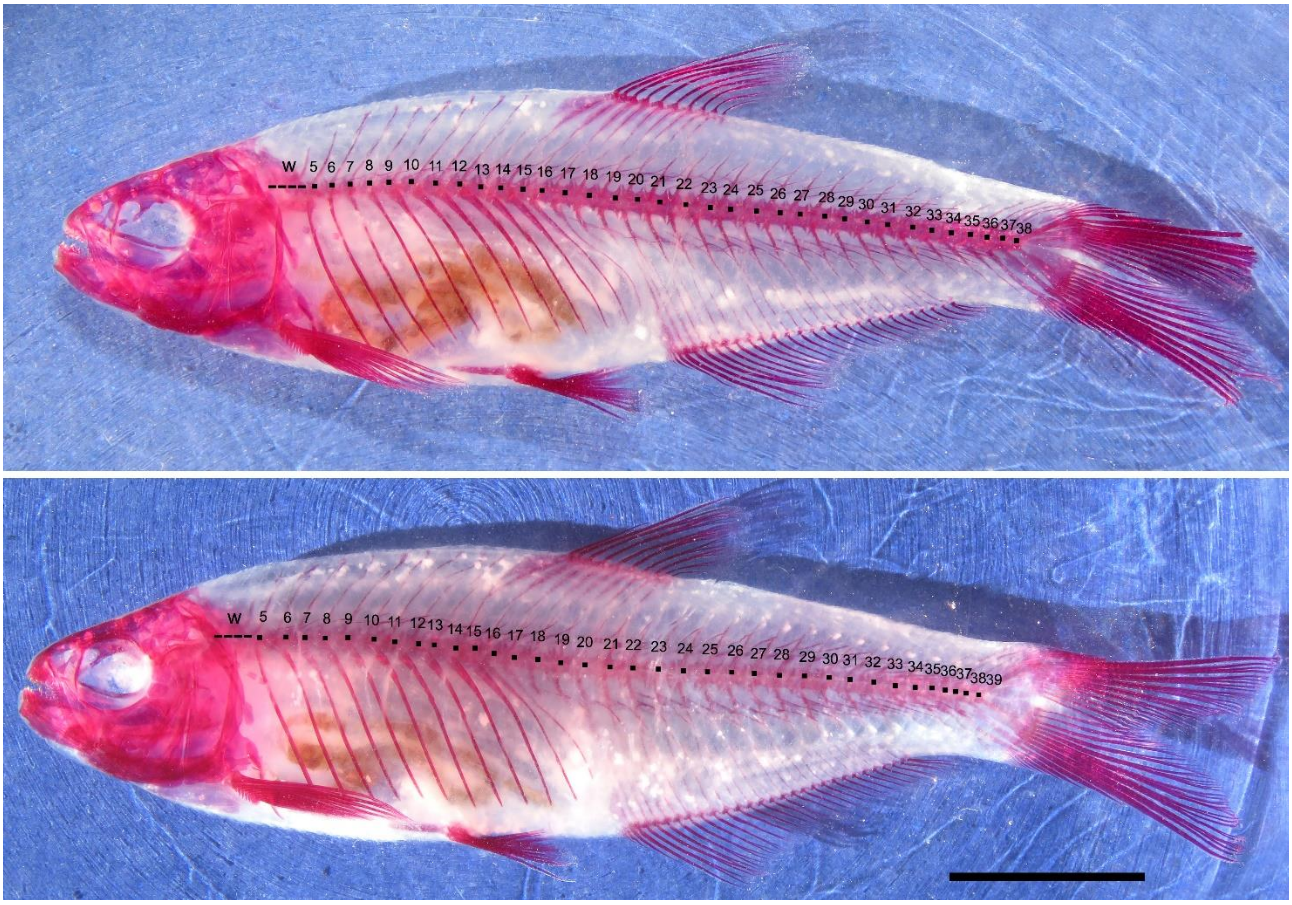

Supplement: Supplementary material 1 — Molecular and taxonomic evidence unmask hidden species diversity in the genus Acrobrycon (Characiformes, Characidae). Fig. S1–S3. [file zookeys-1091-099-s001.pdf]
